# A survey of pathology specimens associated with impacted teeth over a 21-year period

**DOI:** 10.4317/medoral.22873

**Published:** 2019-08-18

**Authors:** Muhanad Mohammed, Farzana Mahomed, Sizakele Ngwenya

**Affiliations:** 1 BDS, MSc(Dent); 2 BChD, MDent, Lecturer, Department of Oral Pathology, School of Oral Health Sciences, Faculty of Health Sciences, University of the Witwatersrand, Johannesburg, South Africa; 3 BDS, MDent, Head of Department, Department of Oral Pathology, School of Oral Health Sciences, Faculty of Health Sciences, University of the Witwatersrand, Johannesburg, South Africa

## Abstract

**Background:**

To compare the histologic diagnosis of lesions associated with impacted teeth from a South African population with literature data.

**Material and Methods:**

A retrospective cross-sectional survey of tissue specimens associated with impacted teeth that were analyzed in the Department of Oral Pathology (University of the Witwatersrand, South Africa) between 1996 and 2016. Patient age, gender, impacted tooth location and the histologic diagnosis were recorded for statistical analysis.

**Results:**

Odontogenic pathology was diagnosed in 389 (95.6%) specimens while dental follicle comprised 4.4% of tissue submissions. The mean age was 25.3 ±15.2 years with a male predilection (M:F=1.8:1). The 11-20 year age group was mostly affected and the overall frequency of odontogenic lesions reduced significantly with an increase in age (*p*=0.01). Dentigerous cyst (56.5%) and ameloblastoma (14%) were most commonly diagnosed.

**Conclusions:**

This is the first African epidemiologic survey of histologic specimens associated with impacted teeth and shows striking differences in the ratio of pathologic to non-pathologic diagnoses compared to other populations. Locally aggressive odontogenic lesions appear to develop one to two decades earlier in patients from developing countries.

** Key words:**Ameloblastoma, biopsy, dentigerous cyst, histopathology, odontogenic cyst, odontogenic tumor.

## Introduction

The World Health Organization (WHO) classification of odontogenic cysts and tumors comprises several distinct entities (1). Many of these entities, however, share similar radiographic features including presentation in association with an impacted tooth. Despite their similar radiographic features, the therapeutic modalities that are advocated for the different entities can vary considerably making accurate distinction between them of paramount importance (1). Histopathology remains the gold standard for the diagnosis of an odontogenic lesion associated with an impacted tooth and the most frequently diagnosed lesion has been that of a dentigerous cyst (2-4). Although the highest prevalence of cysts and tumors associated with an impacted tooth was seen in the third decade in several studies (2,5,6), a strong relationship between increasing age and pathologically significant lesions associated with impacted teeth has been suggested (2). Further, while there are many radiographic surveys reported in the literature on the prevalence of cysts and tumors associated with impacted teeth, there are few large series studies that have included information specifying the histologic diagnosis of the lesion (2-5). This study aimed to determine the frequency of biopsied lesions associated with impacted teeth, and to compare patient demographics and histologic diagnosis with the overall literature.

## Material and Methods

In this retrospective cross-sectional study, all histopathology reports of tissue specimens associated with one or more impacted teeth that were analyzed by oral pathologists between 1996 and 2016 at the University of the Witwatersrand, South Africa, were studied. Cases where the histologic diagnosis was inconclusive due to inadequate tissue, suboptimal representation of lesional tissue or insufficient clinical data were excluded from the study. Patients with known underlying syndromes were also excluded. Radiographic interpretations were not considered other than confirming that the tooth was impacted. Selection of cases was carried out by the principal investigator (MM) and an oral pathologist (FM). Histologic diagnosis of odontogenic lesions were classified according to the fourth edition of the WHO classification (1). Classification of unicystic ameloblastoma was based on the WHO diagnostic criteria which recognizes three types: luminal, intraluminal and mural (1).

The following data were recorded from the histopathology reports: patient age at diagnosis, gender, impacted tooth location and the histologic diagnosis. To avoid duplication, specimens of the same lesion submitted as an incisional biopsy and subsequently as an excisional specimen were distinguished and filtered out to prevent duplication of the data. When more than one lesion occurred in the same patient, the age at which the first lesion was biopsied was recorded. All data were stratified by age and grouped by decade. The location of the associated impacted tooth was classified using the Universal Numbering System; FDI notation. Based on their tendency for recurrence after simple enucleation, the odontogenic lesions were classified as indolent lesions (rarely recur) or aggressive lesions (propensity to recur) according to the WHO classification (1). This study was approved by the local Research Ethics Committee (Approval No. M170812).

-Data analysis

The association between demographic characteristics, location of the impacted tooth and histologic diagnosis were measured using chi-square or Fisher’s exact test. Binary logistic regression analysis was used to determine the dimension of relationship between the histologic diagnosis and patient demographics. Data were analyzed with Statistical Package for the Social Sciences (SPSS), Stata 14.0 (IBM Corp., Armonk, NY, USA). Statistical significance was set at *p*<0.05.

## Results

**Figure 1:**
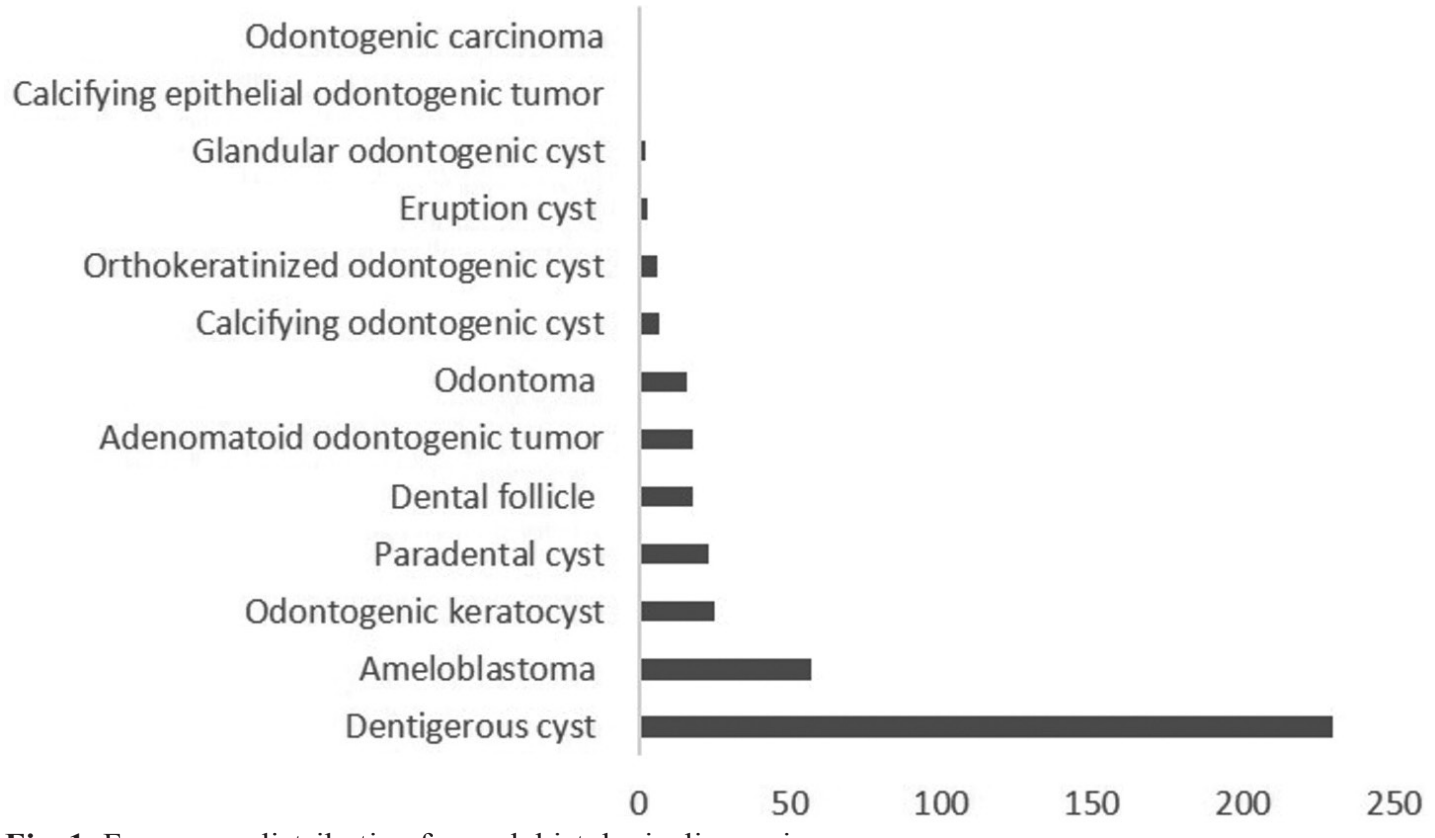
Frequency distribution for each histologic diagnosis.

A total of 24,542 tissue specimens were submitted for histologic examination during the study period; of these 407 (1.7%) were associated with impacted teeth in 390 patients. Tissue specimens were submitted from multiple impacted teeth in 13 (3.3%) patients. The mean age was 25.3 ±15.2 years with a male predilection (n=253; 64.9%). Most patients were 11-20 years in age while the 41-50 year age group comprised the fewest number of patients ( Table 1). Males were significantly more frequently affected than females across all the age groups (*p*=0.01). Figure 1 shows the distribution of the 407 histologic diagnoses. Dentigerous cyst (n=230) and ameloblastoma (n=57) together comprised 70.5% of the diagnoses. Twenty-five (6.1%) odontogenic keratocysts, 23 (5.7%) paradental cysts, 18 (4.4%) adenomatoid odontogenic tumors and 16 (3.9%) odontomas were reported. Cases of calcifying odontogenic cyst (1.7%), orthokeratinized odontogenic cyst (1.5%), eruption cyst (0.7%), glandular odontogenic cyst (0.5%), calcifying epithelial odontogenic tumor (0.2%) and odontogenic carcinoma (0.2%) were also reported. Non-pathologic dental follicle comprised 4.4% of the total number of submissions.

**Table 1:**
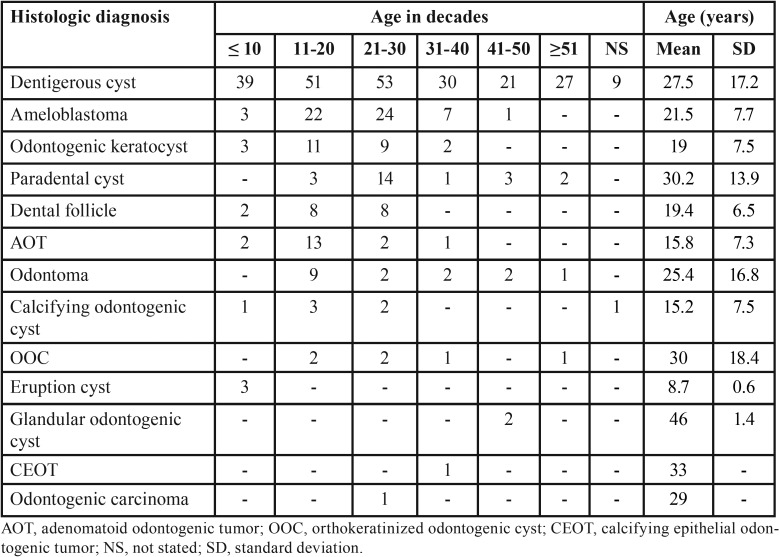
Age distribution and histologic diagnosis of tissue specimens associated with an impacted tooth.

Both dentigerous cyst and ameloblastoma were most frequently diagnosed in the 21-30 year age group. Overall, the frequency of histologic diagnosis for lesions associated with impacted teeth reduced significantly with an increase in age (*p*=0.00) ( Table 1). The ameloblastoma variant was documented in the histopathology report in 48 of the 57 cases. The 20 unicystic ameloblastoma cases comprised eight luminal types, five intraluminal types and seven mural types. The mean age at diagnosis for conventional ameloblastoma was 24.8 years and that of the unicystic type was 17.9 years. For conventional ameloblastoma the male-to-female ratio was almost equal, while the unicystic type was seen twice more commonly in males ( Table 2).

**Table 2:**
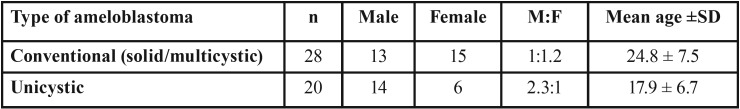
Age and gender distribution of ameloblastoma associated with an impacted tooth.

**Figure 2:**
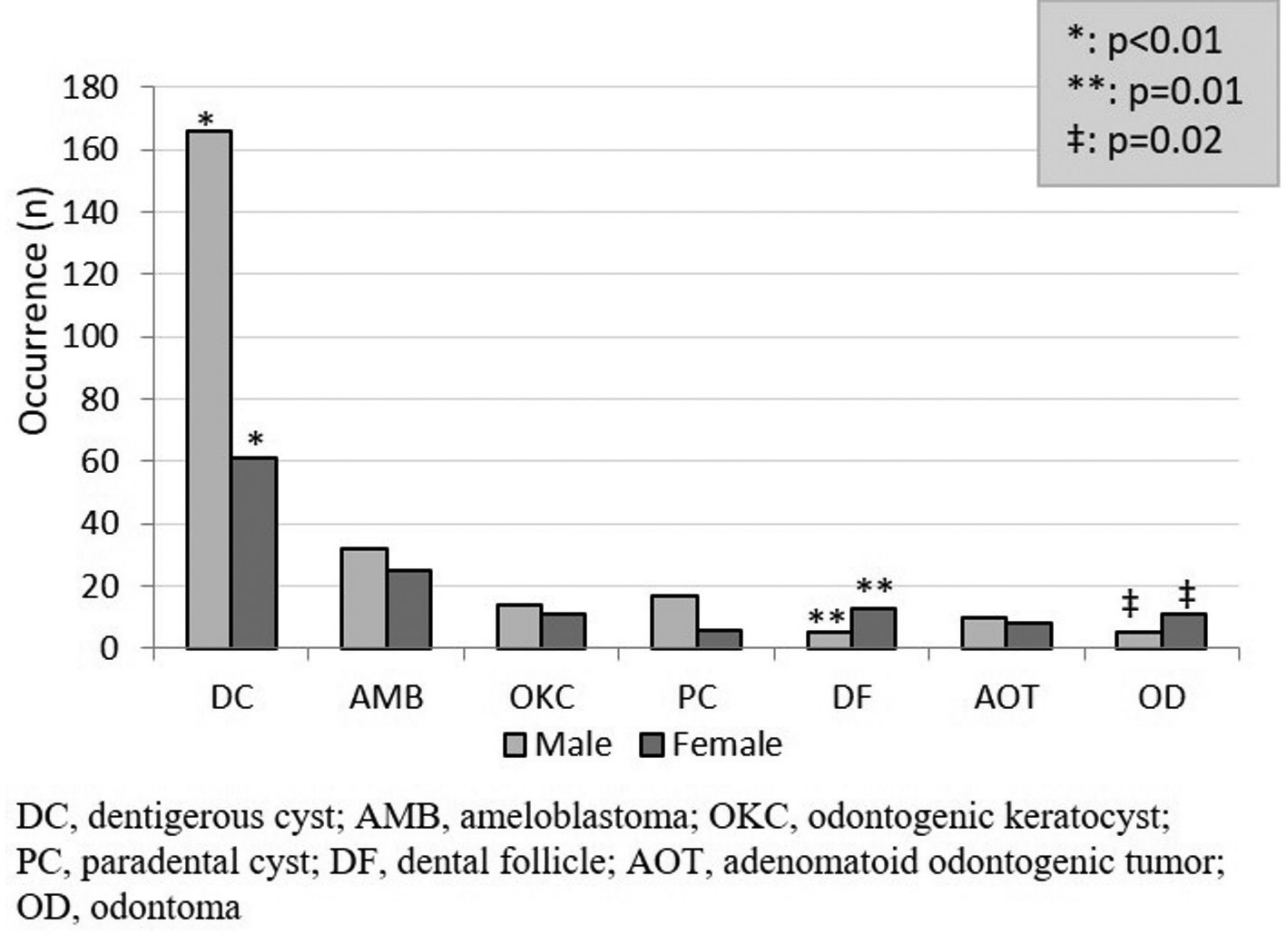
Histologic diagnosis of tissue specimens associated with an impacted tooth according to gender.

Although dentigerous cyst was most commonly diagnosed in the most frequently impacted teeth ( Table 3), no significant association was found between the histologic diagnosis and impacted tooth location (*p*=0.78). Gender showed a significant association with dentigerous cyst, dental follicle and odontoma (Fig. 2). Binary logistic regression analysis showed that dentigerous cyst was diagnosed twice more commonly in male patients (odds ratio [OR], 2.33; 95% confidence interval [CI], 1.53-3.52), while there was a greater than four-fold prevalence of dental follicle (OR, 5.14; 95% CI, 1.95-15.7) and odontoma (OR, 4.25; 95% CI, 1.45-12.49) diagnosed in females. Dentigerous cyst (*p*=0.00), ameloblastoma (*p*=0.00), paradental cyst (*p*=0.01) and adenomatoid odontogenic tumor (*p*=0.03) were significantly more frequently diagnosed in patients during the first three decades of life ( Table 1).

**Table 3:**
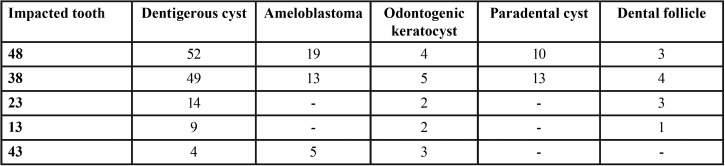
Distribution of the most common histologic diagnoses according to the most frequently impacted teeth.

Odontogenic lesions associated with an indolent biologic behavior showed the highest frequency of histologically diagnosed lesions associated with impacted teeth for all decades studied. These included adenomatoid odontogenic tumor, odontoma, dentigerous cyst, paradental cyst, calcifying odontogenic cyst, orthokeratinized odontogenic cyst and eruption cyst ( Table 1). The following lesions were classified as locally or potentially aggressive lesions; ameloblastoma, odontogenic keratocyst and glandular odontogenic cyst (1). Although ameloblastoma and odontogenic keratocyst were mostly diagnosed in patients in the second and third decade; when comparing indolent with potentially aggressive odontogenic lesions and age the difference did not reach statistical significance (*p*=0.05).

## Discussion

Radiolucencies associated with impacted teeth are commonly encountered in dental practice. A search of the English language literature on the frequency of biopsied jaw lesions associated with an impacted tooth, however, yielded only one previous study (2). In the latter study, biopsy specimens associated with the crown of an impacted tooth represented 7.6 % of the total number of submissions to a diagnostic oral pathology service. By contrast, in the current study 1.7% of tissue specimens were from patients who were radiologically diagnosed with dental follicle, cyst or tumor associated with dental impaction. One of the likely reasons for the seven-fold higher frequency may be related to the fact that 67.1% of the tissue specimens in the study by Curran *et al.* (2) represented dental follicle; while dental follicle comprised only 4.4% of tissue submissions in this study. The pericoronal tissue associated with an impacted tooth is, however, not always submitted for histologic examination and presently there is no universally accepted protocol concerning submission of recoverable soft tissue associated with extracted teeth (7,8), which may account for the comparatively low frequency of dental follicles encountered in our study.

An associated impacted tooth is not an unusual radiographic finding in unicystic and conventional ameloblastoma (9). In a global survey, a mean age of 16.5 years was shown at the time of diagnosis of unicystic ameloblastoma presenting in association with an impacted tooth with a male-to-female ratio of 1.5:1 (10). Similar findings were recorded in the present study with a mean age of 17.9 years for unicystic ameloblastoma although with a higher male-to-female ratio of 2.3:1. The mean age of patients with conventional ameloblastoma associated with an impacted tooth was 24.8 years and was seen slightly more often in females. We sought to determine the frequency of the intraosseous variants of ameloblastoma that presented with an impacted tooth. It was found that most ameloblastomas were of the conventional type (58.3%), followed by unicystic ameloblastoma; luminal type (16.7%), intraluminal type (10.4%) and mural type (14.6%). Future research is needed to clarify the current study finding that most ameloblastomas which are associated with an impacted tooth are of the conventional type. This may have important clinical and therapeutic implications as some reports mention conservative excision with long-term follow-up for the luminal and intraluminal type of unicystic ameloblastoma, while the mural type may behave biologically as conventional ameloblastoma and require local resection of the area (11).

The frequency of odontomas was found to be 3.9%, which corresponds closely to some earlier studies (2,7,8). The relatively low prevalence rates of odontomas in histologic studies may be due to many being diagnosed on radiologic grounds alone. In the present study, 4 of the 7 calcifying odontogenic cysts and 2 dentigerous cysts were associated with odontomas. While the possibility of an odontoma may be suspected on the radiographic findings, the diagnosis of dual or associated pathology can only be determined following histologic examination of the lesion, highlighting the importance of submitting all surgically removed tissue for histologic evaluation. Patients in the second decade showed the highest frequencies of odontomas, odontogenic keratocysts (follicular variant) and adenomatoid odontogenic tumors (follicular variant). This is in keeping with the general trend of age distribution for odontomas and adenomatoid odontogenic tumors but not that of odontogenic keratocysts. In the study by Curran *et al.* (2) 65% of follicular odontogenic keratocysts presented in the fourth decade or later. Our findings thus refute their suggestion that there is a strong relationship between increasing age and the development of pathosis specifically with reference to odontogenic keratocyst.

Odontogenic lesions with an indolent behavior exceeded the potentially aggressive lesions across the first five decades of life, while in patients over the age of 50 years no lesions in the aggressive category were recorded. This finding is probably related to the fact that most lesions in the latter category of this study comprised odontogenic keratocysts and ameloblastoma, which presented at a mean age of 19 years and 21.5 years respectively. In the study by Tsukamoto *et al.* (12) the mean age reported for non-syndromic patients with follicular odontogenic keratocysts was 46.4 years, while in the study by Altini and Cohen (13), 81.2% (13/16) of patients with follicular odontogenic keratocysts were between the ages of 10 and 29 years. While the follicular odontogenic keratocysts in the current study presented more than two decades earlier than in the study by Tsukamoto *et al.* (12), the findings are closer to those of Altini and Cohen (13) who also conducted their study in a South African population. This observation may suggest that geographic variation exists in the demographic profile of patients who present with similar lesions across different parts of the world. Further epidemiologic studies are needed to confirm the observation from the current and previous studies (10,14) that the occurrence of locally aggressive odontogenic lesions associated with impacted teeth present one to two decades earlier in patients from developing countries compared to those from industrialized countries.
